# Supplementary material for: Dynamics of non-household contacts during the COVID-19 pandemic in 2020 and 2021 in the Netherlands
Source: Sci Rep. 2023 Mar 30;13:5166. doi: 10.1038/s41598-023-32031-7 (PMC10060924; doi:10.1038/s41598-023-32031-7)
Supplement: Supplementary file 1 — Supplementary Information. [file 41598_2023_32031_MOESM1_ESM.pdf]

Supplementary information to “Dynamics of non-household contacts during the COVID-19 pandemic in 2020 and 2021 in the Netherlands”

Jantien A. Backer[1,*], Laurens Bogaardt[1], Philippe Beutels[2], Pietro Coletti[3], W. John Edmunds[4], Amy Gimma[4], Cheyenne C. E. van Hagen[1], Niel Hens[3], Christopher I. Jarvis[4], Eric R. A. Vos[1], James Wambua[3], Denise Wong[1], Kevin van Zandvoort[4], and Jacco Wallinga[1,5]

[1]National Institute for Public Health and the Environment, Bilthoven, the Netherlands
[2]University of Antwerp, Antwerp, Belgium
[3]UHasselt, Data Science Institute and I-BioStat, Hasselt, Belgium
[4]London School of Hygiene and Tropical Medicine, London, United Kingdom
[5]Leiden University Medical Center, Leiden, the Netherlands
[*]jantien.backer@rivm.nl

ABSTRACT

The COVID-19 pandemic was in 2020 and 2021 for a large part mitigated by reducing contacts in the general population. To monitor how these contacts changed over the course of the pandemic in the Netherlands, a longitudinal survey was conducted where participants reported on their at-risk contacts every two weeks, as part of the European CoMix survey. The survey included 1659 participants from April to August 2020 and 2514 participants from December 2020 to September 2021.
We categorized the number of unique contacted persons excluding household members, reported per participant per day into six activity levels, defined as 0, 1, 2, 3-4, 5-9 and 10 or more reported contacts. After correcting for age, vaccination status, risk status for severe outcome of infection, and frequency of participation, activity levels increased over time, coinciding with relaxation of COVID-19 control measures.

year	round	0-11	12-17	18-24	25-34	35-44	45-54	55-64	65+	total
2020	target	0	0	162	235	225	277	249	352	1500
2020	1	0	0	88	227	236	308	277	386	1522
2020	2	0	0	43	176	211	277	255	351	1313
2020	3	0	0	31	145	168	246	237	316	1143
2020	4	0	0	30	134	153	223	212	268	1020
2020	5	0	0	35	131	174	247	231	306	1124
2020	6	0	0	21	137	174	233	228	318	1111
2020	7	0	0	20	103	140	215	195	279	952
2020	8	0	0	22	104	142	214	203	284	969
2021	target	150	150	248	206	163	184	161	238	1500
2021	1	137	145	239	202	158	180	158	232	1451
2021	2	126	126	139	175	136	177	176	257	1312
2021	3	107	115	174	175	136	149	128	187	1171
2021	4	99	104	112	157	110	125	142	213	1062
2021	5	81	95	94	127	89	132	127	197	942
2021	6	87	75	110	151	126	118	95	92	854
2021	7	73	67	64	126	85	93	115	143	766
2021	8	68	76	80	133	102	123	78	103	763
2021	9	49	67	78	117	94	86	61	64	616
2021	10	40	65	65	104	89	96	71	31	561
2021	11	116	154	195	205	160	198	167	260	1455
2021	12	107	133	178	194	155	160	142	208	1277
2021	13	96	115	124	163	121	154	152	233	1158
2021	14	84	98	85	143	117	134	140	228	1029
2021	15	77	86	84	135	117	123	113	188	923
2021	16	71	74	50	117	98	117	112	192	831
2021	17	59	82	48	115	103	130	119	106	762
2021	18	55	69	41	90	65	72	105	181	678
2021	19	64	56	43	90	79	73	70	201	676
2021	20	47	81	41	86	84	100	87	160	686

Table S1. Number of participants per survey round of study series in 2020 and 2021 in eight age groups. The target number of participants was to be achieved in round 1 in 2020, and in rounds 1 and 11 in 2021.

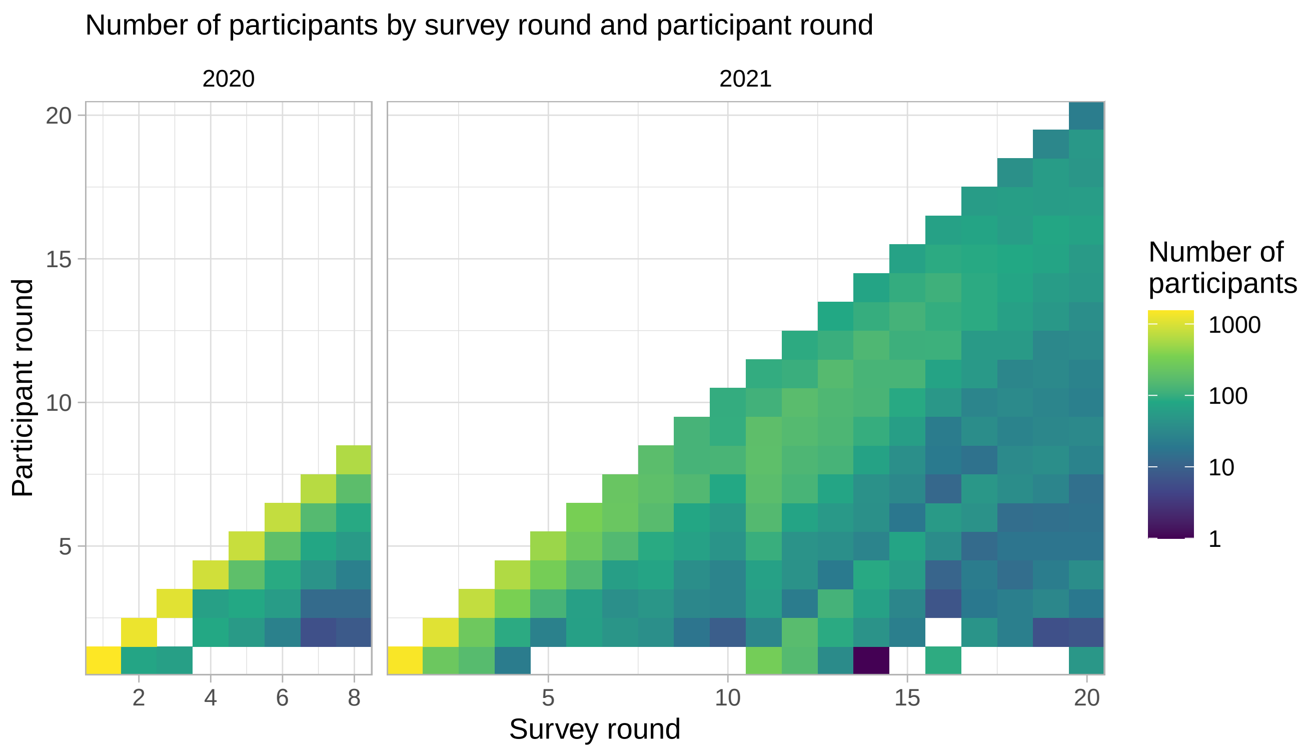

Figure S1. Number of participants by survey round and participant round (i.e. the nth time a participant participated). Series 2020 (left) consisted of 8 rounds, series 2021 (right) consisted of 20 rounds. Most participants participated for the first time the start of the series, but some also started during the study period. The panel was supplemented to the target population in round 11 of series 2021.

series	age_group	Variable	Estimate	Std. Error	t value	p-value	
2020	18-64	(Intercept)	-0.23	0.06	-3.85	< 0.001	***
2020	18-64	part_vaccTRUE	0.00	0.00			
2020	18-64	part_elevated_riskTRUE	-0.03	0.08	-0.40	0.69	
2020	18-64	weekendTRUE	-0.21	0.06	-3.67	< 0.001	***
2020	18-64	holidayTRUE	0.07	0.10	0.72	0.47	
2020	18-64	Deviance explained	0.16				
2020	65+	(Intercept)	0.03	0.13	0.27	0.79	
2020	65+	part_vaccTRUE	0.00	0.00			
2020	65+	part_elevated_riskTRUE	0.25	0.12	2.05	0.04	*
2020	65+	weekendTRUE	-0.32	0.08	-3.89	< 0.001	***
2020	65+	holidayTRUE	-0.08	0.11	-0.70	0.48	
2020	65+	Deviance explained	0.14				
2021	0-17	(Intercept)	-0.81	0.08	-9.91	< 0.001	***
2021	0-17	part_vaccTRUE	0.12	0.19	0.64	0.52	
2021	0-17	part_elevated_riskTRUE	-0.15	0.27	-0.56	0.57	
2021	0-17	weekendTRUE	-0.67	0.08	-8.35	< 0.001	***
2021	0-17	holidayTRUE	-0.20	0.10	-2.04	0.04	*
2021	0-17	Deviance explained	0.17				
2021	18-64	(Intercept)	-0.92	0.06	-16.00	< 0.001	***
2021	18-64	part_vaccTRUE	0.09	0.07	1.31	0.19	
2021	18-64	part_elevated_riskTRUE	-0.13	0.08	-1.72	0.08	.
2021	18-64	weekendTRUE	-0.27	0.05	-5.88	< 0.001	***
2021	18-64	holidayTRUE	0.06	0.06	0.94	0.35	
2021	18-64	Deviance explained	0.19				
2021	65+	(Intercept)	-0.82	0.15	-5.51	< 0.001	***
2021	65+	part_vaccTRUE	0.08	0.19	0.40	0.69	
2021	65+	part_elevated_riskTRUE	-0.10	0.11	-0.89	0.37	
2021	65+	weekendTRUE	-0.44	0.08	-5.65	< 0.001	***
2021	65+	holidayTRUE	0.04	0.10	0.42	0.68	
2021	65+	Deviance explained	0.16				

Table S2. Results for fixed effects of generalised additive model, by series (2020, 2021) and age group (0-17, 16-64, 65+). Included fixed effects are participant vaccination status (part_vacc), participant risk status (part_elevated_risk), weekend and holiday. Holidays include general holidays and school holidays. The last row of each section refers to the explained deviance. Significance levels: *** (p-value < 0.001), ** (p-value < 0.01), * (p-value < 0.05), . (p-value < 0.1)

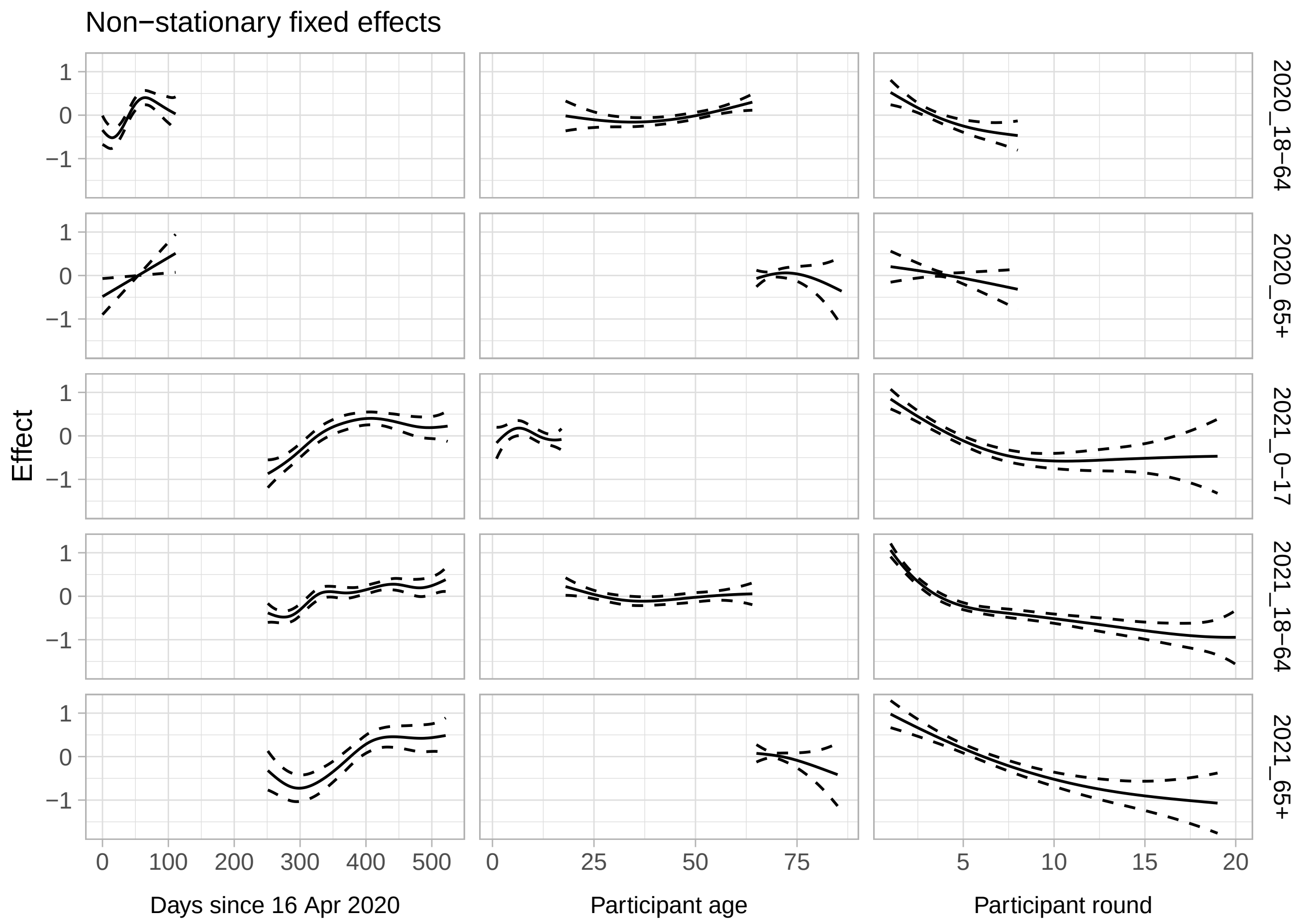

Figure S2. Results for non-stationary fixed effects of generalised additive model, by series (2020, 2021) and age group (0-17, 16-64, 65+). Calendar time (expressed as days since first survey), participant age, and participant round (i.e. the nth time a participant participated) are included as cubic splines. Shown are the fitted splines (solid lines) ± the standard error (dashed lines).

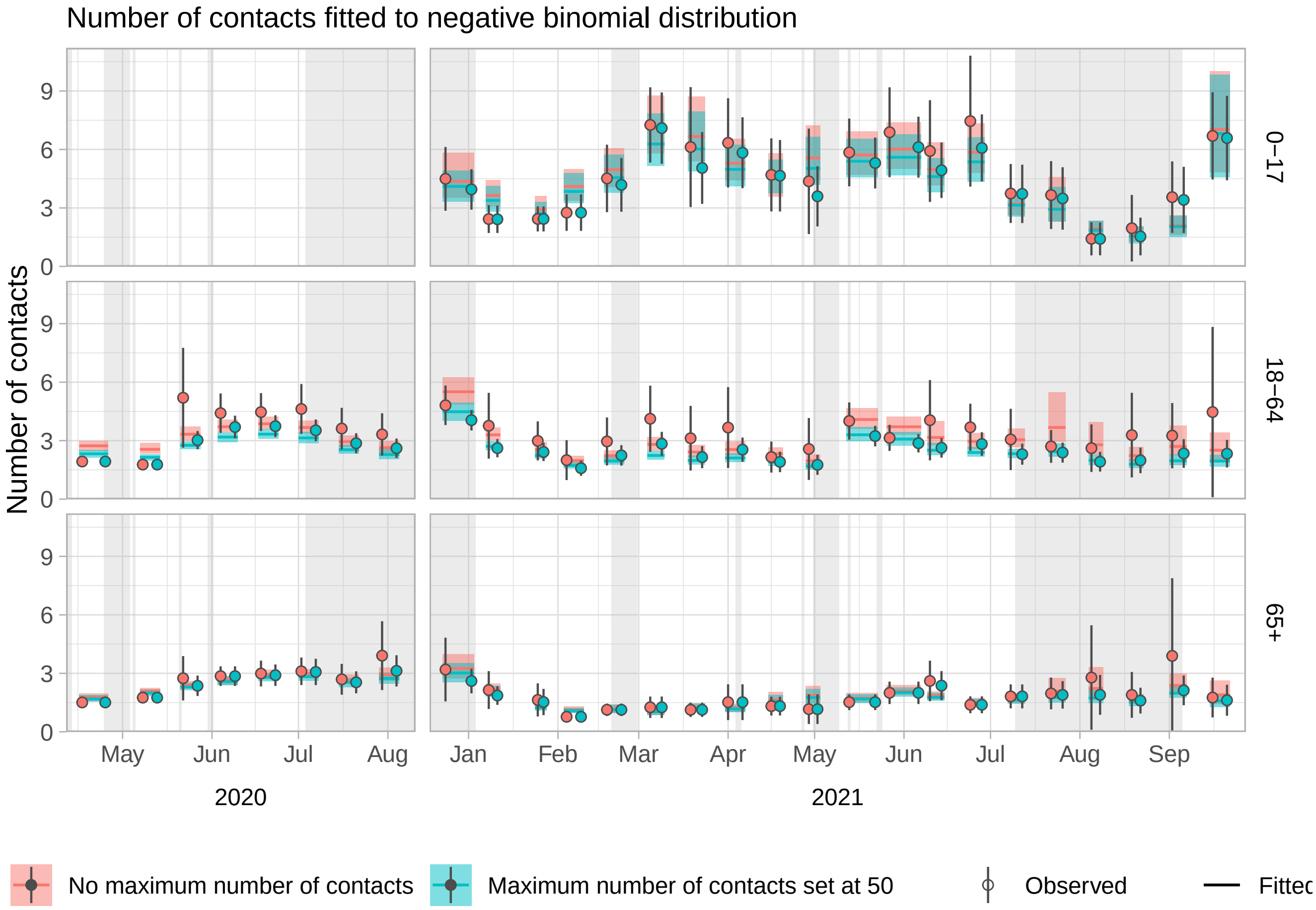

Figure S3. Fitted and observed number of contacts over time by series (columns) and age group (rows), with and without setting a maximum for the number of contacts. The model fits per round are shown by the median (lines) and 95% interval (shaded), from the first to last participation date of the survey round. The mean observed number of contacts, without and with set maximum are placed at the start and end of the survey round (circles, with 95% CI errorbars). Holidays and school holiday periods are shaded in grey. Only 0.8% of the observations are higher than 50, but they lead to much higher and more uncertain estimates. Setting a maximum yields the same trends in contact behaviour as our results but also disregards the heavy tails of the distribution of number of contacts.

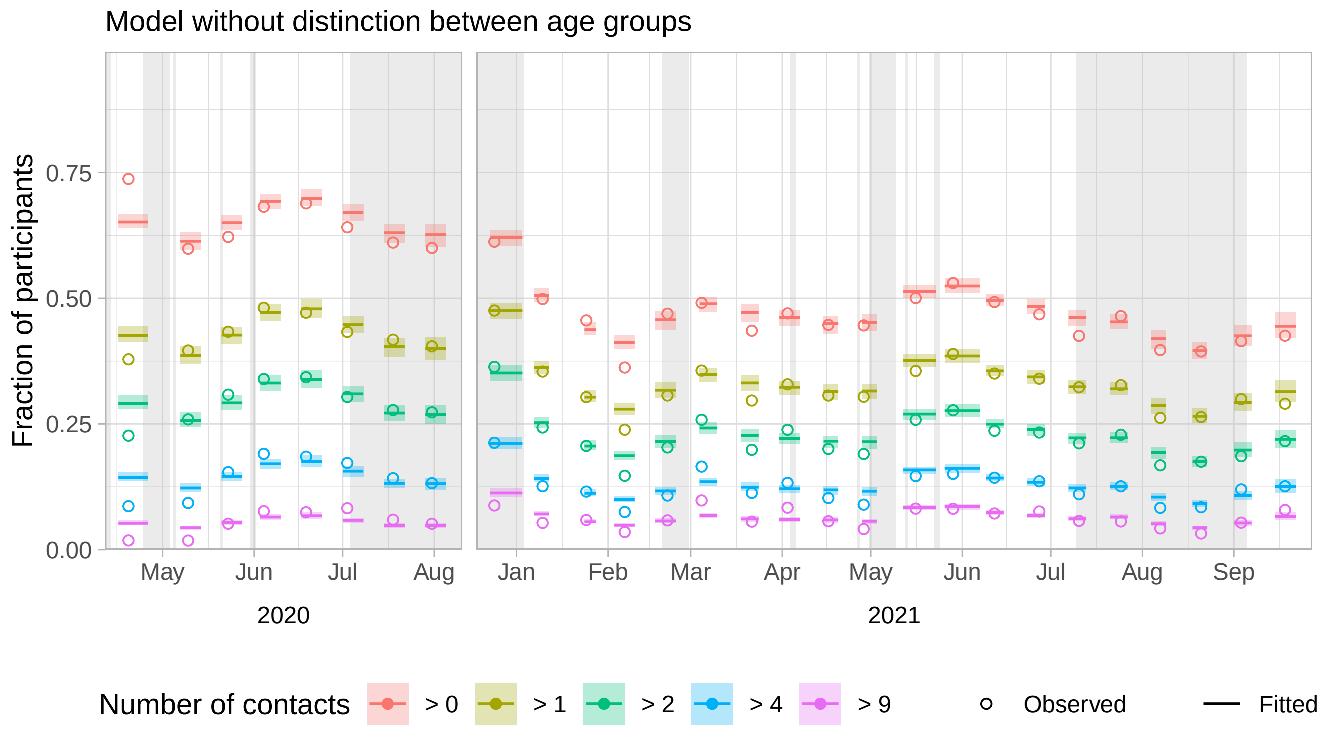

Figure S4. Fitted and observed activity levels over time by series. Activity levels are shown as the fraction of participants that report more than a certain number of non-household contacts per day. With five limits (>0, >1, >2, >4 and >9) six activity levels are defined, e.g. the fraction between the limits of >2 and >4 is the activity level that represents 3 or 4 contacts per participant. The model fits per round are shown by the median (lines) and 95% interval (shaded), from the first to last participation date of that survey round. The observed activity levels per round (open circles) are placed at the mean participation date of that survey round. Holidays and school holiday periods are shaded in grey. The model presented in the main text distinguishes between 3 age groups (0-17, 18-64, and 65+), while here they are analysed together. Participant age is modelled with a spline over the full age range. Although not markedly different from the main results, this analysis fails to capture any interactions between age group and calendar time, for instance when the oldest age group increased their contacts in 2021 later than the rest of the population (see Fig. S2).
